# A High Copper Concentration Copper-Quadrol Complex Electroless Solution for Chip Bonding Applications

**DOI:** 10.3390/ma17071638

**Published:** 2024-04-03

**Authors:** Jeng-Hau Huang, Po-Shao Shih, Vengudusamy Renganathan, Simon Johannes Gräfner, Yu-Chun Lin, Chin-Li Kao, Yung-Sheng Lin, Yun-Ching Hung, Chengheng Robert Kao

**Affiliations:** 1 Department of Materials Science and Engineering, National Taiwan University, Taipei 106, Taiwan; f07527007@ntu.edu.tw (J.-H.H.); f08527055@ntu.edu.tw (P.-S.S.); renganathank.v.r@gmail.com (V.R.); sim.gra@hotmail.de (S.J.G.); typs94040@gmail.com (Y.-C.L.); 2 Advanced Semiconductor Engineering Group, Kaohsiung 811, Taiwan; golden_kao@aseglobal.com (C.-L.K.); viktor_lin@aseglobal.com (Y.-S.L.); megan_hung@aseglobal.com (Y.-C.H.)

**Keywords:** 3D integration, electroless Cu plating, low-temperature bonding, pressure free bonding, Taguchi method, copper–quadrol complex solution

## Abstract

This article presents a novel bonding method for chip packaging applications in the semiconductor industry, with a focus on downsizing high-density and 3D-stacked interconnections to improve efficiency and performance. Microfluidic electroless interconnections have been identified as a potential solution for bonding pillar joints at low temperatures and pressures. However, the complex and time-consuming nature of their production process hinders their suitability for mass production. To overcome these challenges, we propose a tailored plating solution using an enhanced copper concentration and plating rate. By eliminating the need for fluid motion and reducing the process time, this method can be used for mass production. The Taguchi approach is first used to optimize the copper–quadrol complex solution with the plating rate and decomposition time. This solution exhibits a copper concentration that is over five times higher than that of conventional solutions, a plating rate of 22.2 μm/h, and a decomposition time of 8 min on a Cu layer substrate. This technique enables Cu pillars to be successfully bonded within 7 min at 35 °C. Planarizing the pillar surface yields a high bonding percentage of 99%. Mechanical shear testing shows a significant fracture strength of 76 MPa.

## 1. Introduction

Over the past few decades, the semiconductor industry has increasingly focused on scaling down high-density and 3D-stacked interconnections, owing to their high efficiency and superior performance. However, the downsizing of these components presents new challenges and requires innovative chip packaging. Although the most prevalent technologies currently used in this field include hybrid bonding interconnections, solid–liquid interfacial diffusion, and anisotropic conductive films, all these methods share the common drawback of high pressures and temperatures. These disadvantages can have adverse effects on the surrounding delicate parts [1,2,3,4]. Nonetheless, electroless plating is a promising and viable solution for future chip packaging applications [5].

Electroless deposition (ELD) is a popular technique used to prepare thin films of metals and their alloys. Compared to other physical and chemical vapor deposition methods, this method offers several benefits, such as low-temperature, autocatalytic processes, high selectivity, and low cost. Consequently, ELD has been extensively employed in various applications ranging from the production of electronic circuits and interconnections to micro/nanoscale technology [6,7]. The first use of an electroless-plated deposition layer to connect facing pillar bumps was reported by He et al. [8]. Subsequently, (Hung et al., 2021) developed a new technique called microfluidic electroless interconnection (MELI). This method employs electroless plating to form highly uniform interconnections on a single-chip scale [9].

Researchers have investigated the characteristics of various metal ions such as Cu, Ni-P, and Au. To achieve wafer-level packaging and 3D integration, they also applied 2-inch wafer-level scale chips and dual chips. Additionally, they utilized numerical fluidic-chemical multiphysics simulations to investigate plating conditions and mechanisms under different flow regimes [9,10,11,12]. However, the MELI process, which employs a polydimethylsiloxane (PDMS) flow channel, a syringe pump, and electroless plating, is not feasible for mass production in the current industry because of its complexity and time-consuming nature. The procedure involves creating a microfluidic fixture using patterned PDMS and a glass plate, followed by the injection of an electroless plating solution into the microchannel using a syringe pump. This bonding process takes approximately 1 h to complete for Cu pillar interconnections, which is too long for practical industrial applications [9,10]. Therefore, the objective of this study is to identify a potential solution to this issue.

Huang et al. developed a copper–glycerin complex solution with a high copper concentration, specifically for use in electroless plating [13]. This solution had a significantly higher plating rate than commercially available electroless Cu plating solutions and a Cu concentration that was more than four times higher than that of conventional solutions. By combining a high-concentration copper–glycerin complex solution and bonding technology, Huang et al. developed a straightforward, low-temperature, time-efficient, and pressureless process for bonding copper pillars. By using this solution, the copper pillars could be successfully bonded without requiring an external flow. The processing time was reduced to 9 min, and the operating temperature was lowered to 29 °C, thereby making it more suitable for industrial applications. This method presents a promising solution to the challenges posed by the MELI process, such as the long processing time and the need for a syringe pump [14]. However, this study did not include a significant number of bonded joints, and the plating rate of this formula remained insufficient. Therefore, the present study employs the Taguchi method to develop a new solution formula to enhance the plating rate, and the interconnections are designed using a surface planar to improve the bonding percentage of the joints.

In general, a basic electroless copper plating bath comprises a metal ion source, reducing agents, complexing agents, and stabilizers; it is carried out at a controlled temperature and pH. Among the copper salts, copper sulfate is the most widely used and can be obtained with a purity of 99.5% [15]; thus, it was selected as the preferred copper salt in this study. Formaldehyde is commonly used as a reducing agent in commercial electroless copper solutions because of its cost-effectiveness and ease of control [16]. Therefore, formaldehyde was used in the present study. To enhance the deposition rate of the plating solution effectively, a quadrol was chosen as the complexing agent. According to a previous study, this ligand has a higher plating rate than glycerin and is more stable than other complexes, such as tartrate complexes [17]. Therefore, the main goal of this study is to develop a copper–quadrol complex solution with a high copper concentration. A previous patent presented a high copper concentration copper–quadrol complex solution with a Cu concentration of 0.2 M, which is over five times that of conventional electroless copper plating solutions [18]. Nonetheless, a high Cu concentration poses difficulties such as self-decomposition and a short decomposition time, making it difficult to use in the process [13]. As a result, the concentrations of copper sulfate, formaldehyde, and surfactant were kept constant in this study, whereas the concentrations of quadrol, cytosine, temperature, and pH were varied to optimize the solution.

Although numerous techniques have been developed for process optimization, the Taguchi approach has been widely adopted in engineering design because of its ease of use and applicability to users with limited statistical knowledge. The Taguchi method utilizes a unique orthogonal array design to analyze the entire parameter space with a minimal number of experiments. In this study, a three-level factorial design with four factors was used. Using an appropriate orthogonal array from the Taguchi method, the study was conducted with only nine experimental runs, reducing the total number of trials from 81 to 9 [19,20,21]. It was assumed that there was no interaction between the operating factors. The two main factors investigated in this study were plating rate and decomposition time. A plating solution with a high plating rate and an appropriate decomposition time yielded better results. The weight method is commonly used to measure the solution plating rate, as stated in references [16,22]. There is no standard method for evaluating the stability of a plating solution in terms of decomposition time. Based on the literature, a simple way to determine the stability of the solution is to observe the deposition of Cu particles and the formation of H_2_ bubbles [22,23], and the decomposition time was determined using these two factors. Mean effect studies were conducted to investigate the impacts of the plating rate and decomposition time, and the parameters were ranked in order of importance. The study then demonstrated the application of the bonding technology with a high-concentration copper–quadrol complex solution. The correlation between the average difference in pillar height and the bonding percentage of the pillars was investigated. Furthermore, the analysis included an examination of the joints, the morphological characteristics of the cross sections, and the evaluation of mechanical properties. Finally, this study successfully developed a new process with a low temperature (<100 °C) and no pressure, which completely solves the problems associated with high pressures and temperatures in existing technologies [1,2,3,4].

## 2. Materials and Methods

### 2.1. Optimization of Copper–Quadrol Complex Solution

#### 2.1.1. Taguchi Method

Table 1 outlines the composition of the electroless plating bath and plating conditions [18]. The four most dominant parameters were identified as the concentrations of quadrol, cytosine, temperature, and pH. To design the experiments, the Taguchi method was employed, with each factor divided into three levels to consider nonlinear effects, as presented in Table 2. An L_9_ orthogonal array (OA) was selected based on the degrees of freedom, and the design factors and their respective levels were allocated to the resulting matrix, where each row represented a single test in the experimental setup [19,20,21,24]. The plating rate and decomposition time were measured in each experiment. By analyzing the optimal set of parameters, this method can predict the response of each factor, which is effective not only in experimental or confirmatory tests but also in a single set of experimental results [25,26,27,28]. In this method, engineering judgment is commonly used to optimize multi-response problems. Engineers rely on their expertise and experience to determine the optimal levels of these factors [28,29,30]. Although ensuring the quality of this method is challenging, it saves time and money. In this study, the control factors were determined separately, and their respective optimum levels were identified for each response variable. Subsequently, tradeoffs were made to optimize the formulation of the plating solution.

#### 2.1.2. Sample Preparation and Coating Characterization

**Figure 1:**
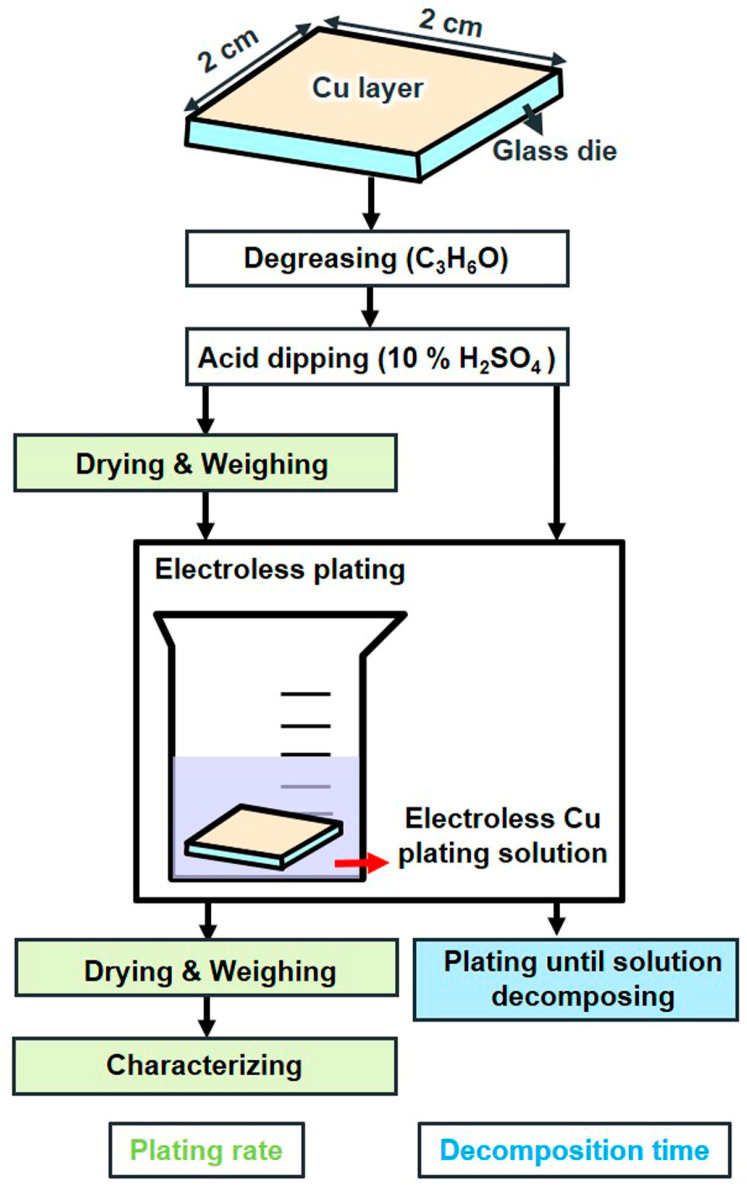
Flow chart of the pre-treatment.

For this study, a glass substrate with a thickness of 0.5 mm was used, and a 30 nm Cr adhesion layer and a 200 nm Cu seed layer were consecutively sputtered onto it. The wafers were then sliced into individual dies measuring 2 cm × 2 cm. The pre-treatment processes are shown in Figure 1. First, the sample was degreased through ultrasonication for 1 min using acetone, and the copper (II) oxide was cleaned with 10% H_2_SO_4_ for 1 min. Two different approaches were employed to examine the plating rate and decomposition time of the plating solution. The plating rate was determined using the weight method described below:

v = 10,000 Δm/ρSt(1)

where v denotes the plating rate in μm/h, Δm is the mass increment of the sample after plating in g, ρ represents the copper density (8.9 g/cm^3^), S denotes the area of the sample in cm^2^, and t denotes the plating time in h [22]. The sample was first dried and weighed before plating, then immersed in the plating solution to initiate the reaction. Once the plating solution decomposed, the plating process was immediately terminated. The plating rate was then determined by drying and weighing the sample using Equation (1). The decomposition time was assessed by monitoring the emergence of H_2_ bubbles and the deposition of Cu particles in the plating solution with visible light [13]. Once one of the phenomena occurred, we recorded the time and considered it the decomposition time.

### 2.2. Concept Testing

#### 2.2.1. Fabrication of the Test Vehicle

First, a 30 nm Cr adhesion layer was deposited onto a 4 in glass wafer, followed by a 200 nm Cu seed layer. Then, a 15 μm dry film photoresist layer (RY-3315EE, manufactured by Hitachi Chemical, Tokyo, Japan) was hot-rolled onto the wafer. The photoresist was patterned by UV light exposure and developed in a 1% sodium carbonate solution. After patterning, the wafer underwent electroplating to produce pillars with a height of 17 ± 2 μm. The pillar diameter was approximately 50 μm, with a pitch of 100 μm. Finally, the wafer was diced into 8 mm × 8 mm dies.

**Figure 2:**
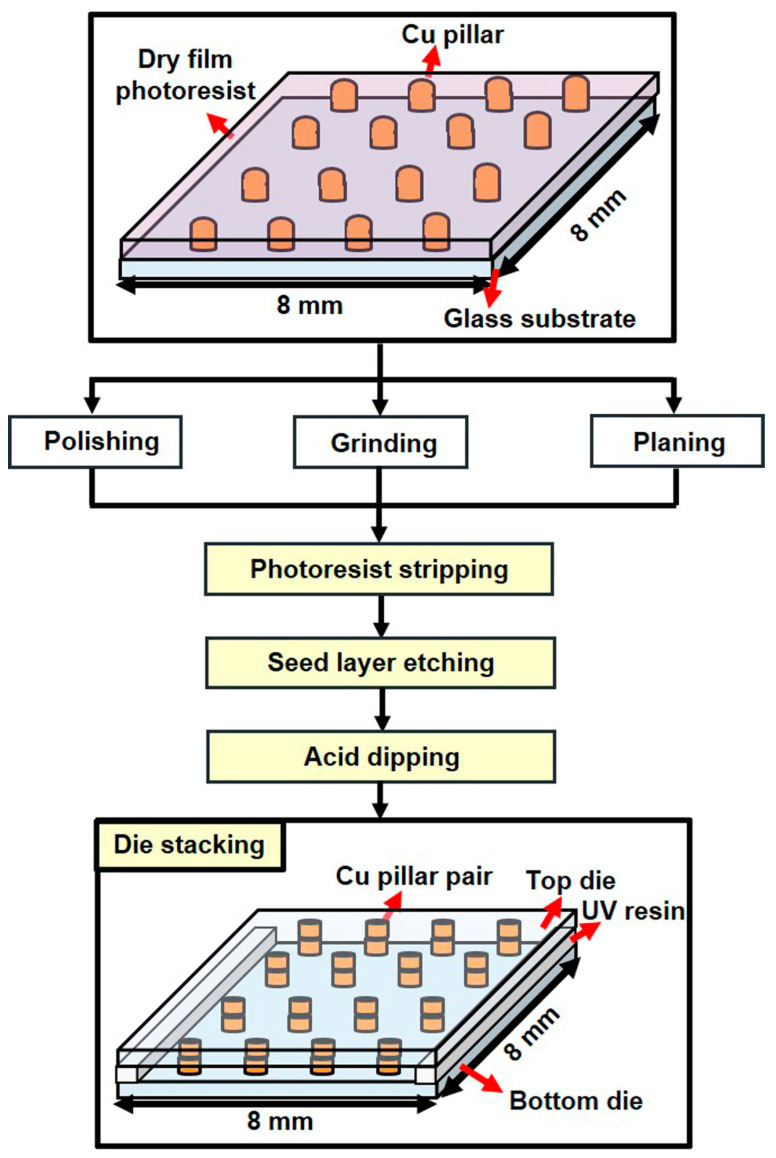
Flow chart of test vehicle fabrication.

To prepare the dies for electroless plating, a pre-treatment process involving multiple steps was required. The first step involved the use of different methods to flatten the pillars and determine optimal results. These methods included sandpaper polishing, machine grinding (SpeedFam, BSG-VFT0436, Hsin Chu, Taiwan), and surface planar planning (Disco, DFS8910, Tokyo, Japan). The die was then soaked in a 10% sodium hydroxide solution to remove the photoresist layer. The Cu and Cr layers were selectively etched using sodium persulfate (SPS) and hydrochloric acid. To remove the copper oxide from the surface of the copper pillars, the die was immersed in 10% sulfuric acid. Next, a layer of UV resin was applied to one die, and another die with a matching pillar pattern was aligned on top using a flip-chip die bonder (Tresky, T-3002-FC2, Thalwil, Switzerland). After exposing the UV resin to UV light and hardening it, a test vehicle was established, and the electroless plating process began. A flow chart of the test vehicle fabrication process is shown in Figure 2. The pre-bonded pillar height in this study ranged from approximately 16–36 μm, depending on the flattening process. The gap between the pillars was approximately 1–6 μm.

#### 2.2.2. Electroless Cu Plating Process

**Figure 3:**
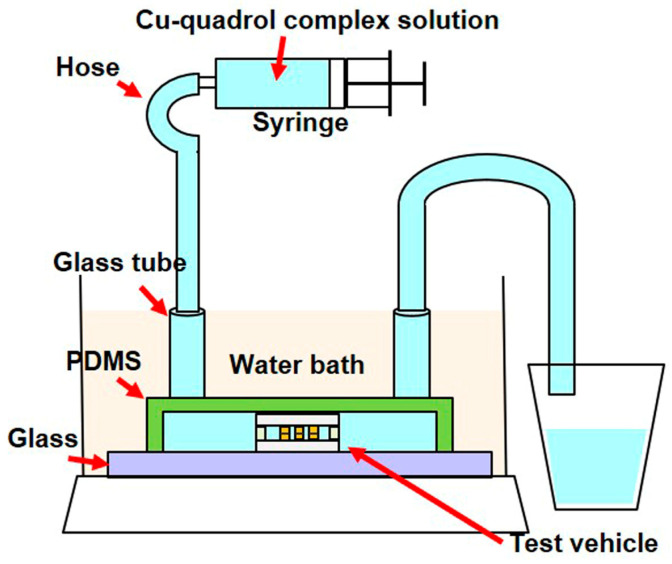
Schematic of the experiment.

A PDMS-based die mold was first fabricated to verify the plating conditions in the die, and then the test vehicle was placed in the PDMS die mold. The optimized copper–quadrol complex solution was injected into the microchannel and maintained in a stationary state during plating. Subsequently, the device was immersed in a thermostatic bath at 35 °C for 7 min. A schematic of the experiment is shown in Figure 3. After completing the electroless Cu plating process, the die was removed from the PDMS die mold and separated by dissolving the UV resin in acetone. The morphology of the bonded Cu pillars was examined using a scanning electron microscope (SEM), while the cross-sectional analysis of the electroless-plated Cu was performed using a focused ion beam (FIB). In addition, the fracture strength was obtained using a bond tester (XYZTC, Condor Sigma, Hsin Chu, Taiwan).

## 3. Results and Discussion

### 3.1. Effect of Plating Parameters on Plating Rate and Decomposition Time

This study aims to investigate the effects of four variables (quadrol, cytosine, temperature, and pH) on the plating rate and decomposition time of the plating solutions. The results are presented in Table 3, which shows that the highest plating rate of 22.2 μm/h and decomposition time of 8 min were achieved in test 7. This plating rate is greater than that of a commercial electroless Cu plating solution (5 μm/h) and the copper–glycerin complex solution (8 μm/h) on an open surface [13]. Table 4 and Table 5 display the main-effect response tables for the plating rate and decomposition time, respectively. The delta value corresponds to the variation between the highest and lowest mean plating rates and decomposition times for each variable level. The larger the delta value, the more significant the corresponding factor becomes. Based on the ranking positions, cytosine had the highest impact on the plating rate, followed by pH, temperature, and quadrol. In addition, pH was the most significant factor affecting decomposition time, followed by temperature, cytosine, and quadrol.

#### 3.1.1. Effect of the Concentration of Quadrol in the Solution

**Figure 4:**
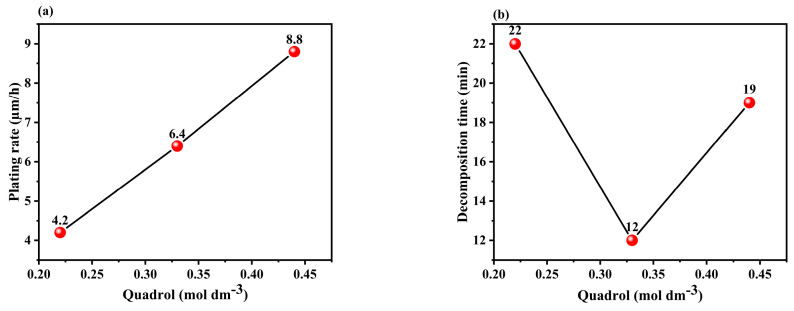
Effect plot of quadrol concentration vs. (**a**) plating rate and (**b**) decomposition time.

Quadrol acted as a complexing agent, improving the plating rate and bath stability. The reaction between quadrol and Cu^2+^ facilitates the reduction of Cu, leading to a significant increase in the deposition rate (as shown in Equation (2)) [31]. Additionally, the formation of a cuprous complex prevents side reactions (as shown in Equations (3) and (4)) [32], which stabilizes the plating bath and extends the decomposition time. However, there is a slight deviation in the quadrol-to-decomposition time curves, indicating that other factors, such as bath composition, may have a minor impact on the complexing effects [27]. Figure 4a,b shows that the highest plating rate and optimum decomposition time were achieved at a concentration of 0.44 M quadrol.

Cu(II)-L + 2HCHO + 4OH^−^ → Cu^0^ + H_2_ + 2HCOO^−^ + 2H_2_O + L (2)

2Cu^2+^ + HCHO + 5OH^−^ → Cu_2_O + HCOO^−^ + 3H_2_O (3)

Cu_2_O + H_2_O → Cu^0^ + Cu^2+^ + 2OH^−^ (4)

where L: ligand.

#### 3.1.2. Effect of the Concentration of Cytosine in the Solution

**Figure 5:**
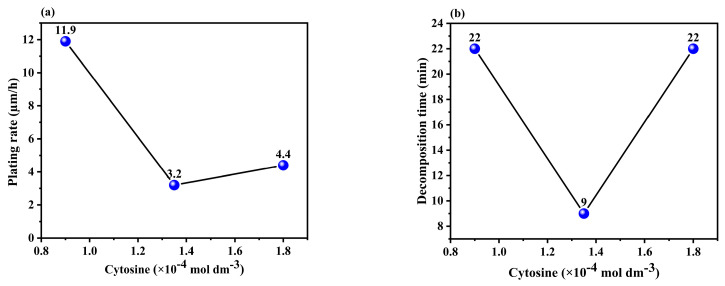
Effect plot of cytosine concentration vs. (**a**) plating rate and (**b**) decomposition time.

Cytosine served as both a stabilizer and an accelerating agent in the plating process. A stabilizer prevents the plating bath from decomposing, whereas an accelerating agent increases the deposition rate. Figure 5a shows that a small amount of cytosine significantly increased the plating rate, which is consistent with the findings of previous studies [16,18]. The acceleration of the plating rate with the inclusion of cytosine can be attributed to the existence of delocalized π-electron bonds in its structure, which promotes the plating process. This was because of the higher adsorption capacity of cytosine at low concentrations on the reactive copper surface, which facilitated the reduction of copper ions and accelerated the deposition rate. However, excessive copper surface coverage can decrease the deposition rate [16,33]. Figure 5b illustrates that the bath life can be effectively extended by adding a small amount of organic additives, as mentioned in the literature [16]. However, larger amounts of additives did not significantly affect the decomposition time and may have even decreased the plating rate. Therefore, the optimal concentration of cytosine is 9 × 10^−5^ M.

#### 3.1.3. Effect of the Temperature of the Solution

**Figure 6:**
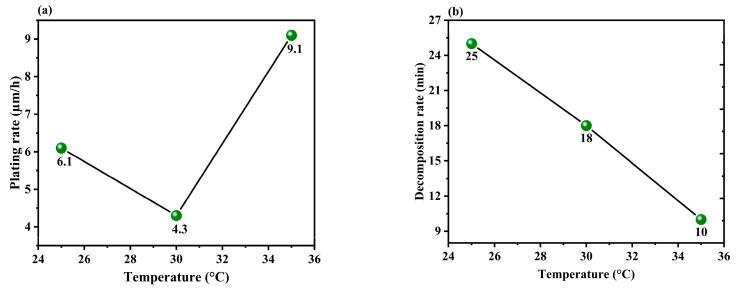
Effect plot of temperature vs. (**a**) plating rate and (**b**) decomposition time.

Copper–quadrol complex solutions are typically used at elevated temperatures ranging from 35 to 55 °C, although formulations that can be used at room temperature are also available [34]. Nonetheless, to prevent the plating solution from decomposing, the experiments in this study were carried out within the temperature range of 25 to 35 °C. The effect of temperature on both plating rate and decomposition time is presented in Figure 6a,b, which shows that as the bath temperature increases, the plating rate also increases significantly up to 35 °C, confirming the positive effect of temperature observed in similar copper coating experiments [35,36]. The underlying mechanism can be described by the basic Arrhenius rate equation (Equation (5)), which includes parameters such as A (pre-exponential factor), ΔE (activation energy), and C_MG_^−^ (methylene glycol anion concentration resulting from formaldehyde hydration) [37].

R = A exp(−ΔE/RT) C_MG_^−^ (5)

The exponential factor in the equation was responsible for the substantial increase in the plating rate, even with a slight temperature difference. The slight deviation in the relationship between temperature and plating rate can be attributed to the minor complexing effects due to other concentration variables. Moreover, an increase in the operating temperature accelerates the decomposition reactions described in Equations (3) and (4), leading to a significant reduction in decomposition time. Since the objective of this study is to effectively enhance the plating rate while ensuring that the decomposition time is sufficient for operation, the optimal operating temperature is determined to be 35 °C.

#### 3.1.4. Effect of the pH of the Solution

**Figure 7:**
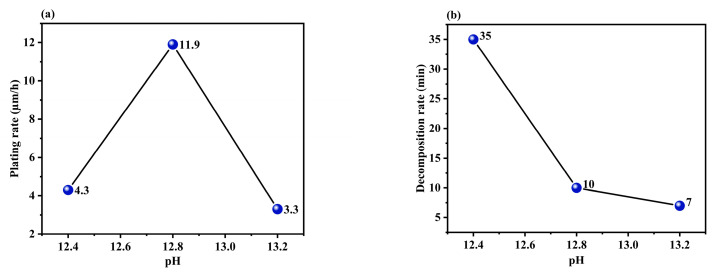
Effect plot of pH vs. (**a**) plating rate and (**b**) decomposition time.

Figure 7a,b shows the plating rate and decomposition time plotted at various pH levels. The plating rate gradually increased with an increase in the pH of the solution. This is because higher hydroxide concentrations typically lead to an increase in the catalytic oxidation of HCHO, which in turn results in a higher plating rate, as described by Equation (2) [38]. However, a sudden decrease in the plating rate occurred beyond a certain pH, which is a phenomenon observed in other copper coatings [36]. This is because a high pH can cause the plating solution to decompose, thereby reducing the decomposition time, copper ion concentration, and plating rate. Finally, the experiments conducted in this study showed that a pH of 12.8 produced the optimum performance in terms of plating rate and decomposition time.

In summary, this study found that the optimal bath composition for achieving a proper plating rate and decomposition time consists of 0.2 M copper sulfate, 0.27 M formaldehyde, 0.44 M quadrol, 9 × 10^−5^ M cytosine, 0.001 g/L PLURONIC F-127, 35 °C, and a pH of 12.8. The concentration of copper was enhanced by more than five times compared to that in a typical electroless copper plating solution. This formulation also demonstrated a high plating rate (22.2 μm/h) and an appropriate decomposition time (8 min) on a 2 cm × 2 cm Cu layer substrate.

### 3.2. Plating Condition of Electroless Cu Plating Process

#### 3.2.1. Morphology of Bonded Cu Pillars

**Figure 8:**
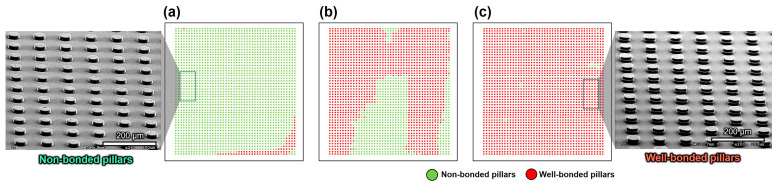
Schematic of the bonding results using copper–quadrol complex solution at 35 °C for 7 min with copper pillar after (**a**) polishing, (**b**) grinding, and (**c**) surface planing.

The optimized copper–quadrol complex solution was injected into the test vehicle and allowed to react at 35 °C for 7 min. To avoid solution decomposition, the reaction time was kept shorter than the decomposition time. After the completion of the reaction, the sample was dissolved in acetone, and the two dies were separated to observe the bonded pillars. Subsequently, we discuss the bonding outcomes with different copper pillar surface treatments, as illustrated in Figure 8. As shown in Figure 8a, only 3% of the Cu pillar pairs were bonded (red pillars) after polishing with sandpaper and electroless plating with copper–quadrol complex solution at 35 °C for 7 min. The rest were considered non-bonded pillars (green pillars), and the SEM image displayed their morphologies. The bonding percentage was low because some copper pillar pairs in the test vehicle had large gaps owing to the high average pillar height difference. Therefore, only pillar pairs with small gaps could be bonded, whereas the other pillar pairs with large gaps were left unbonded. Detailed information is provided in Table 6.

**Figure 9:**
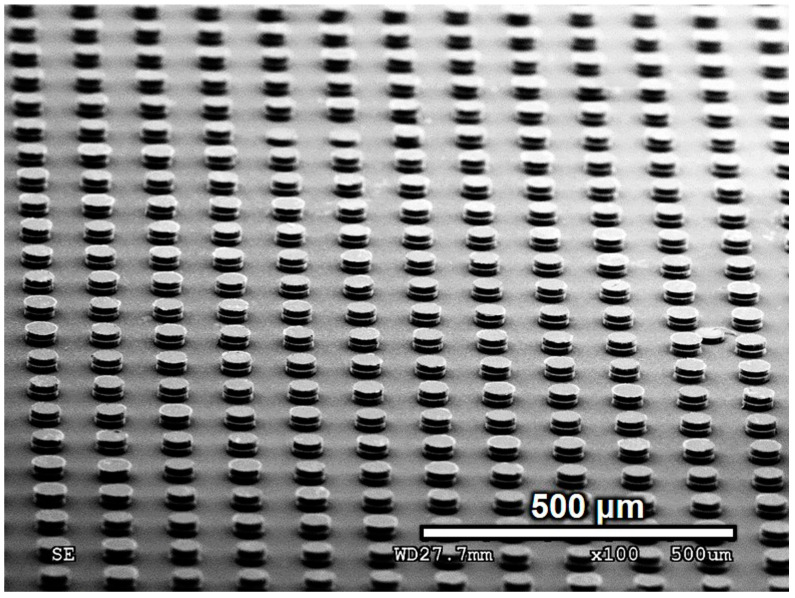
Micrograph showing the bonded pairs of Cu pillars.

The average pillar height was determined by measuring three samples using a 3D laser scanning microscope. The average pillar height difference is defined as the difference between the maximum and minimum average pillar heights. No copper pillar pairs were observed to have bonded when the average pillar height difference was 3.6 μm. However, when the pillar height difference was reduced to 3 μm, the bonding percentage increased to 3%. Figure 8b shows the results of the copper pillars after grinding. The bonding percentage significantly improved to 70% when the pillar height difference was 2.2 μm. After using the surface planar to plan the pillar surface, the average pillar height difference decreased to 0.4 μm. Moreover, as shown in Figure 8c, the bonding percentage reached 99%, and the SEM image shows the morphology of the well-bonded Cu pillars. Therefore, the average pillar height difference is a crucial factor in achieving a large number of bonded joints. With a smaller difference in the pillar height, the bonding percentage increased significantly. Figure 9 shows that 99% of the copper pillar array was successfully bonded after electroless Cu plating at 35 °C for 7 min. A few non-bonded pillars were formed owing to the weak adhesion layer, causing the pillars to break down before bonding. Most of the bonded copper pillars remained attached because of the stronger bonding strength between the pillar pairs compared to the adhesion layer. If the bonding strength is weaker than that of the adhesion layer, the pillars will fracture during the separation of the two dies.

#### 3.2.2. Cross Section of Electroless-Plated Cu

**Figure 10:**
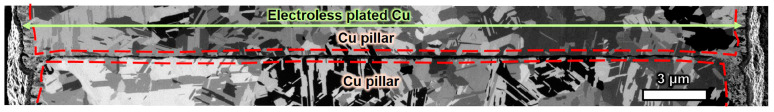
Cross-sectional micrograph showing the bonded copper pillars.

Figure 10 shows a cross-sectional view of the bonded pillars following a 7 min electroless Cu plating process at 35 °C. The surfaces of the electroplated and electroless-plated copper showed a distinct variation in grain size. The growth of electroless-plated copper around the edges of the pillars hindered the movement of the copper ions toward the center, causing voids between the pillars. Similar issues have been highlighted in previous studies [9,39] and can be resolved by adopting dome-shaped pillars instead of flat-topped pillars [9].

#### 3.2.3. Mechanical Property Measurements

**Figure 11:**
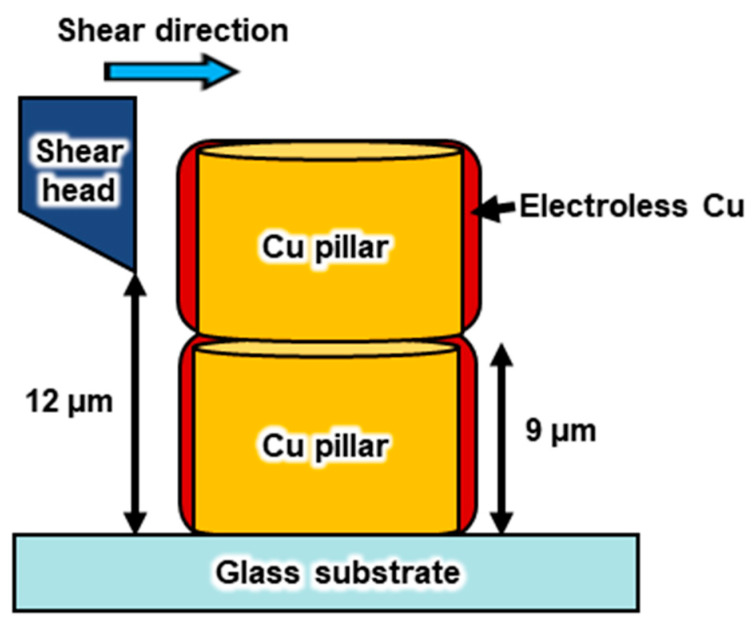
Schematic of the shear test.

To assess the bond strength of the joined connections, a bond tester was used to conduct tests on 16 samples of bonded pillar pairs. The shear speed was 10 μm/s, and the shear height was set at 12 μm above the substrate, positioned in proximity to the upper region of the joint, as illustrated in Figure 11. The results revealed a 100% fracture at the jointed section, specifically at the interface between the electroless Cu-plated layer and the Cu pillar. The average fracture strength of the electroless Cu bonds was measured at 76 MPa, surpassing the values reported for electroless copper bonding in the literature (18–36 MPa) [40]. Additionally, it slightly exceeded the fracture strengths of SAC305 (59 MPa) and SnPb (65 MPa) solder joints [41]. These findings underscore the robustness of the electroless-Cu-bonded interconnections utilizing the copper–quadrol complex solution, indicating their suitability for practical applications.

#### 3.2.4. Comparison of this Method with Existing Technologies

Finally, we compared this method with existing technologies. First, in contrast to hybrid bonding, it eliminates the need for an annealing process to facilitate copper expansion for contact, thereby reducing thermal stress. Moreover, the plating solution is transported without the application of pressure, unlike hybrid bonding, which necessitates high pressure to connect copper surfaces. Second, compared to solid–liquid interfacial diffusion, this method does not involve thermal compression bonding, thereby mitigating the risk of chip warpage due to high pressure and temperature. Third, in comparison to anisotropic conductive films, the bonding temperature is lower, even approaching room temperature. Additionally, it obviates the requirement for high pressure to redistribute particles. Based on these considerations, we underscore the significance of this process [2,3,4].

## 4. Conclusions

In this study, a novel bonding method with a straightforward approach, low-temperature, short processing time, and pressureless nature was developed. This investigation introduces a bonding approach that utilizes an optimized plating solution characterized by a high copper concentration and an increased plating rate. By eliminating the reliance on fluid flow, this method has enhanced its potential for mass production. The Taguchi approach was utilized to optimize the rate of plating and duration of decomposition by employing an L_9_ orthogonal array comprising four main-effect parameters at three levels each. Main-effect response tables and graphs were used to analyze the impact of each parameter. Cytosine was identified as a critical factor influencing the plating rate, whereas pH significantly affected the decomposition time.

The optimized copper–-quadrol complex solution was obtained with the following parameters: 0.2 M copper sulfate, 0.27 M formaldehyde, 0.44 M quadrol, 9 × 10^−5^ M cytosine, 0.001 g/L PLURONIC F-127, 35 °C, and pH = 12.8. The copper concentration in the electroless copper plating solution increased by more than five times compared to that in conventional solutions, revealing the key factors for optimizing a high copper concentration solution. This formulation exhibited a substantial plating rate of 22.2 μm/h and a suitable decomposition time of 8 min on a 2 × 2 cm Cu layer substrate. By applying this solution, Cu pillars could be successfully bonded within 7 min at 35 °C. The relationship between the average difference in pillar height and the bonding percentage of the pillars was further examined. A bonding percentage of 99% was attained by planarizing the pillar surface. In addition, comprehensive investigations were conducted on the joint and cross-sectional morphologies. Despite the presence of a significant void in the center of the bonded pillar, this issue can potentially be addressed by implementing dome-shaped pillars in future applications. The mechanical shear test yielded a bond strength of 76 MPa, exceeding the typical strengths found in commonly used solder joints and other electroless Cu-plated joints. In summary, the combination of an optimized plating solution with a high copper concentration and an enhanced plating rate, together with the chip bonding method, presents an extremely promising approach for future applications in 3D integration. Furthermore, this very-low-operating-temperature and pressureless process demonstrates significant potential for use in 3D integration compared to existing technologies.

## 5. Future Works

In this study, there are additional research avenues worthy of exploration. These include investigating the interactions between different factors and assessing the long-term reliability of the bonded joints. These aspects will be delineated in future research endeavors.

## Figures and Tables

**Table 1:** Composition of electroless plating bath and plating conditions.

Bath Parameters	Value
Copper sulfate	0.2 (mol dm^−3^)
Formaldehyde	0.27 (mol dm^−3^)
PLURONIC F-127	0.001 g/L
Quadrol	0.22–0.44 (mol dm^−3^)
Cytosine	0.9–1.8 × 10^−4^ (mol dm^−3^)
Temperature	25–35 °C
pH	12.4–13.2

**Table 2:** Design factors and their levels.

Design Factors	Levels		
	1	2	3
Quadrol (mol dm^−3^)	0.22	0.33	0.44
Cytosine (×10^−4^ mol dm^−3^)	0.9	1.35	1.8
Temperature (°C)	25	30	35
pH	12.4	12.8	13.2

**Table 3:** Experimental results from L_9_ OA.

					Results	
Test. No.	A: Quadrol (mol dm^−3^)	B: Cytosine (×10^−4^ mol dm^−3^)	C: Temperature (°C)	D: pH	Plating Rate (μm/h)	Decomposition Time (min)
1	0.22	0.9	25	12.4	7	52
2	0.22	1.35	30	12.8	4.2	7
3	0.22	1.8	35	13.2	1.5	8
4	0.33	0.9	30	13.2	6.5	6
5	0.33	1.35	35	12.4	3.5	12.5
6	0.33	1.8	25	12.8	9.3	16
7	0.44	0.9	35	12.8	22.2	8
8	0.44	1.35	25	13.2	1.9	8
9	0.44	1.8	30	12.4	2.3	41

**Table 4:** Main-effect response table for plating rate.

Level	Factor			
	Quadrol	Cytosine	Temperature	pH
1	4.2	11.9	6.1	4.3
2	6.4	3.2	4.3	11.9
3	8.8	4.4	9.1	3.3
Delta	4.6	8.7	4.8	8.6
Rank	4	1	3	2

Unit: μm/h.

**Table 5:** Main-effect response table for decomposition time.

Level	Factor			
	Quadrol	Cytosine	Temperature	pH
1	22	22	25	35
2	12	9	18	10
3	19	22	10	7
Delta	10	13	15	28
Rank	4	3	2	1

Unit: min.

**Table 6:** Bonding results with different sample pre-treatments.

	Average Pillar Height	Average Pillar Height Difference	Bonding Percentage
Original sample	15.6–19.2 μm	3.6 μm	0%
Sample after polishing	14.3–17.3 μm	3 μm	3%
Sample after grinding	8–10.2 μm	2.2 μm	70%
Sample after surface planing	8.5–8.9 μm	0.4 μm	>99%

## Data Availability

Data are contained within the article.
